# Trabecular bone in the calcaneus of runners

**DOI:** 10.1371/journal.pone.0188200

**Published:** 2017-11-15

**Authors:** Andrew Best, Brigitte Holt, Karen Troy, Joseph Hamill

**Affiliations:** 1 Department of Anthropology, University of Massachusetts, Amherst, Massachusetts, United States of America; 2 Department of Biomedical Engineering, Worcester Polytechnic Institute, Worcester, Massachusetts, United States of America; 3 Department of Kinesiology, University of Massachusetts, Amherst, Massachusetts, United States of America; University of Colorado Boulder, UNITED STATES

## Abstract

Trabecular bone of the human calcaneus is subjected to extreme repetitive forces during endurance running and should adapt in response to this strain. To assess possible bone functional adaptation in the posterior region of the calcaneus, we recruited forefoot-striking runners (n = 6), rearfoot-striking runners (n = 6), and non-runners (n = 6), all males aged 20–41 for this institutionally approved study. Foot strike pattern was confirmed for each runner using a motion capture system. We obtained high resolution peripheral computed tomography scans of the posterior calcaneus for both runners and non-runners. No statistically significant differences were found between runners and nonrunners or forefoot strikers and rearfoot strikers. Mean trabecular thickness and mineral density were greatest in forefoot runners with strong effect sizes (<0.80). Trabecular thickness was positively correlated with weekly running distance (r^2^ = 0.417, p<0.05) and years running (r^2^ = 0.339, p<0.05) and negatively correlated with age at onset of running (r^2^ = 0.515, p<0.01) Trabecular thickness, mineral density and bone volume ratio of nonrunners were highly correlated with body mass (r^2^ = 0.824, p<0.05) and nonrunners were significantly heavier than runners (p<0.05). Adjusting for body mass revealed significantly thicker trabeculae in the posterior calcaneus of forefoot strikers, likely an artifact of greater running volume and earlier onset of running in this subgroup; thus, individuals with the greatest summative loading stimulus had, after body mass adjustment, the thickest trabeculae. Further study with larger sample sizes is necessary to elucidate the role of footstrike on calcaneal trabecular structure. To our knowledge, intraspecific body mass correlations with measures of trabecular robusticity have not been reported elsewhere. We hypothesize that early adoption of running and years of sustained moderate volume running stimulate bone modeling in trabeculae of the posterior calcaneus.

## Introduction

Trabecular bone forms porous networks in long bone epiphyses, joint articulations, and throughout the internal volumes of the foot bones. Elastic properties of trabecular bone are a function of several characteristics, including the average thickness of each trabecular strut (trabeculae), the number of struts per unit area, the total volume of trabecular bone per unit area (bone volume fraction: a function of trabecular number and thickness), orientation and connectivity of struts, and mineral density. Density of trabecular bone was first correlated with mechanical properties 50 years ago [1, 2], and by the 1970’s and 1980’s architectural properties were as well [3, 4, 5, 6]. Computed tomography, an x-ray imaging tool, now allows for precise and nondestructive analysis of trabecular architecture. Diederichs et al. [7] found that CT-derived trabecular properties are highly predictive of mechanical strength in the calcaneus, particularly bone mineral density (r^2^ = 60%), bone volume fraction, (r^2^ = 63%) and average thickness of trabeculae (r^2^ = 53%). Trabeculae are usually aligned with the principle direction of strain [8], and degree of anisotropy- a measure of trabecular alignment- is positively correlated with mechanical strength in the prevailing direction of trabeculation [9].

The process by which bone adapts to stress was first described by Wolff [10] and others [11] and later refined and termed bone functional adaptation (BFA) by Ruff et al. [12]. While BFA has been debated in its details and mechanisms, it is generally accepted that long bone diaphyseal shape and bending strength respond to loading [12], although some researchers have argued that cross-sectional shape does not reflect loading history (e.g. [13]). Recent work has clearly shown a similar adaptive response in trabecular bone. Animal experiments demonstrate increases in trabecular thickness, mineral density, bone volume fraction, and anisotropy in response to loading [14, 15, 16, 17, 18], and trabecular bone measures are frequently used to infer physical behavior, including locomotor patterns, in hominin and primate skeletal material [19, 20, 21, 22, 23, 24, 25, 26, 27]. Loading-induced increases in trabecular number are seen in young animal experiments [18, 15]; this variable is thought to be malleable early in life and does not comprise a significant part of bone adaptation in adults. However, some evidence suggests that attenuation of trabecular bone volume with ageing differs between the sexes, with women losing trabecular number and men losing trabecular thickness, perhaps leading to increased number of (now thinner) trabeculae in men [28].

The purpose of the present study is to investigate possible trabecular bone adaptation resultant from a common activity that subjects bone to a regime of repetitive and high strain: endurance running. Previous work has identified cortical and trabecular bone adaptation in the tibia of endurance runners [29, 30, 31]. We chose to study the calcaneus, not just because it endures tremendous forces during endurance running but also because its function differs based on foot strike. Foot strike patterns during running include forefoot striking (FFS), where the forefoot makes initial contact; midfoot striking (MFS), where the forefoot and heel make ground contact simultaneously; and rearfoot striking (RFS), where the heel strikes first before the body’s center of mass moves forward over the forefoot. Given these differences the calcaneus may reflect not just adaptation to endurance running, as has been suggested by recent research [32] but also to the specific and differential demands of each foot strike.

Trabecular structure in the human calcaneus is complex and varies by anatomical region. The highest trabecular thickness and bone volume ratio is found just inferior to the talo-calcaneal joint while the posterior region has the highest trabecular number [33]. Several networks of trabeculae intersect in the posterior region: compressive trabeculae extend from the subtalar articular surface inferiorly and posteriorly; tensile trabeculae run roughly perpendicular to the compressive network, extending from the calcaneal tuberosity to the anterior plantar surface; and tendotuberosity trabeculae abut and run parallel to the tuberosity [34]. During running, forces in the calcaneus begin with impact and increase through the stance phase as the soleus and gastrocnemius generate tension on the Achilles tendon. Forces increase with running velocity and far exceed those during walking [35, 36]; as such, bone adaptation is likely partly a function of running speed [31]. Giddings et al [37] reported that calcaneal loading peaks at 60% of the stance phase, and estimated the following forces at 3.71 m/s: 11.1 body weights at the talo-calcaneal joint; 7.9 body weights at the cuboid-calcaneal joint; and the 7.7 body weights at the Achilles tendon insertion. This study appears to have been conducted with rearfoot-striking runners and the influence of foot strike may be significant. Talo-calcaneal forces may be greater in RFS as the calcaneus is the first point of ground contact, and conversely, Achilles tendon forces acting on the calcaneus are greater in FFS. Forefoot strikers experience Achilles tendon loading earlier in the stance phase than RFS (at initial ground contact) and these forces peak 19% higher (6.3 body weights vs. 5.1 body weights) than RFS [38]. Thus, forces acting on at least two regions of the calcaneus differ based on foot strike.

We hypothesized that trabecular bone of the posterior calcaneus would show evidence of adaptation resultant from forces associated with endurance running. Specifically, runners should have greater calcaneal trabecular thickness and mineral density that nonrunners; these measures should be positively correlated with running volume and years running, and inversely correlated with age at onset of running; and forefoot runners should have more robust trabecular architecture than rearfoot runners. We did not expect calcaneal degree of anisotropy to differ between test groups as the calcaneus experiences loading in several planes.

## Methods

### Participants

Nineteen healthy males between the ages of 20 and 41 (mean = 29.4; SD = 5.7) were recruited using flyers, emails to athletic organizations, and word of mouth. Participants reported their running habits and history, age, body mass, height, and other exercise habits (Table 1) in a questionnaire. Six participants self-reported as nonrunners and had a wide range of physical activity levels from nearly sedentary to highly active (activities included Crossfit training, weight lifting, recreational bicycling and hiking). Nonrunners reported walking on average between 0.5 and 2 miles per day. Non-runners ranged from 21–41 years of age (mean = 28.7, SD = 6.9) and body mass ranged from 68 to 112 kg (mean = 87.8, SD = 16.4). Thirteen participants self-reported as runners, defined as regularly running at least 40 km per week, with a weekly distance range of 40–105 km (mean = 67.1, SD = 22.9) and an age range of 20–39 years (mean = 29.8, SD = 5.4). Body mass was significantly lower in runners (mean = 72.2, SD = 5.2, p<0.05) but was not significantly different in FFS vs. RFS. Years running ranged from 2–21 (mean = 9.8, SD = 5.6). Age at which runners began habitually running ranged from 11–37 years (mean = 19.9, SD = 7.8). Runners were further subdivided based upon foot strike pattern, either FFS or RFS. Forefoot strikers had an earlier average onset of running, higher average weekly running volume and lower body mass than rearfoot strikers. Forefoot runners had lower body mass than nonrunners (p<0.05). Note that our definition of FFS encompasses a purported third foot strike method, midfoot strike, often defined as simultaneous contact between the heel and forefoot. None of our runners landed simultaneously on the heel and forefoot; any participant who landed first with the forefoot, even by fractions of a second, were categorized as forefoot runners. Study protocol was approved by institutional review boards at the University of Massachusetts Amherst and Worcester Polytechnic Institute and all participants gave written informed consent.

**Table 1:** Subject characteristics.

	FFS	RFS	All Runners	Nonrunners
*N*	6	6	13	6
Age (yrs)	26.5 ±4.7	33.3 ±4.4	29.8 ±5.4	28.7 ±6.9
Body mass (kg)	70.4 ±3.8*	75.0 ±5.4	72.2 ±11.5*	87.8 ±16.4
Weekly running distance (km)	79.2 ±25.1	58.3 ±16.6	67.1 ±22.9	n/a
Years running	10.5 ±6.1	9.2 ±6.0	9.8 ±5.6	n/a
Age at onset of running	16.0 ±4.3	24.2 ±9.2	19.9 ±7.8	n/a

Subject characteristics (mean ±sd).

*Significantly different than nonrunners (p<0.05).

### Assessment of foot strike

Runners participated in an initial data collection at the University of Massachusetts Amherst Biomechanics Laboratory. Foot strike was confirmed by asking participants to run down a 20m runway at preferred “average” training pace, shod, while affixed with tracking markers. Data were collected using high-speed motion capture cameras and a single force plate which recorded ground reaction forces for the right foot. Slow-motion playback confirmed the foot strike pattern. One of these participants self-reported that his foot strike varies with pace, and this was confirmed with visual examination on a treadmill. As such his CT data were only included in runner vs. nonrunner analysis. Six runners were categorized as FFS and six as RFS.

### Quantification of trabecular bone measures

Scans were obtained using high resolution peripheral computed tomography, or HRpQCT (Xtreme CT; Scanco Medical AG, Bassersdorf, Switzerland), by Karen Troy and Joshua Johnson at Worcester Polytechnic Institute. Scan settings included the following: Effective energy- 60 kVp; x-ray tube current- 0.9 mA; matrix size- 1536 x 1536; filter- 0.3 mm Cu and 1 mm Al; voxel size—82 μm. We measured the following trabecular variables: volumetric bone density (DTrab; of trabecular bone only) in mg hydroxyapatite per cm^3^; trabecular bone volume/total volume (BV/TV); mean thickness of trabecular struts (Tb.Th); average number of trabeculae per unit length (Tb.N); average distance between trabeculae (Tb.Sp); and degree of anisotropy (DA), a measure of trabecular alignment, defined as length of the longest divided by the shortest mean intercept length vector [39]. Structural measures obtained from HR-pQCT hold up well when compared to the “gold standard” μCT, which has superior resolution [40]. However, Tb.Th is an exception. It is worth briefly discussing this issue. Voxel size for the scanner we used is 82 μm, near the size of individual trabeculae. Given this limitation, the standard Scanco analysis (which we used) employs an indirect method to assess trabecular thickness, detailed by Laib et al [41] and Laib and Ruegsegger [42]. In short: A derived bone volume ratio (BV/TV^d^) is calculated by dividing the measured trabecular bone density (DTrab) by 1200 mg HA cm^3^ (the assumed density of fully mineralized bone; see MacNeil and Boyd, 2007 for a discussion of this limitation). Trabecular number is measured directly using a method that has proven accurate [43]. Trabecular thickness is calculated as BV/TV^d^ /Tb.N instead of using a direct measurement method, thus minimizing the limitation created by a voxel size near that of individual trabeculae. Measuring Tb.Th with HR pQCT using this semi-derived method is now commonplace and several studies have quantified the repeatability and precision of this method. Boutroy et al [44] measured Tb.Th in the distal tibia (Tb.Th = 76 μm) and distal radius (Tb.Th = 89 μm) 3 times each in 15 individuals repositioned for each scan. A coefficient of variation (CV) was calculated as the standard deviation of the 3 measures divided by the subject mean. The resulting CV value for Tb.Th was 4.4%. MacNeil and Boyd [40] found only a modest correlation between Tb.Th measures obtained from HR-pQCT and μCT (r^2^ = 0.59) suggesting that the former may not reliably measure absolute values. However, precision was excellent: HR-pQCT Tb.Th values were consistently lower than those from μCT, a phenomenon that Scanco themselves have acknowledged [44], but a regression slope of 1.0 indicates near perfect precision. Thus, HR-pQCT using the semi-derived method is useful for comparing relative Tb.Th of samples or individuals.

**Fig 1:**
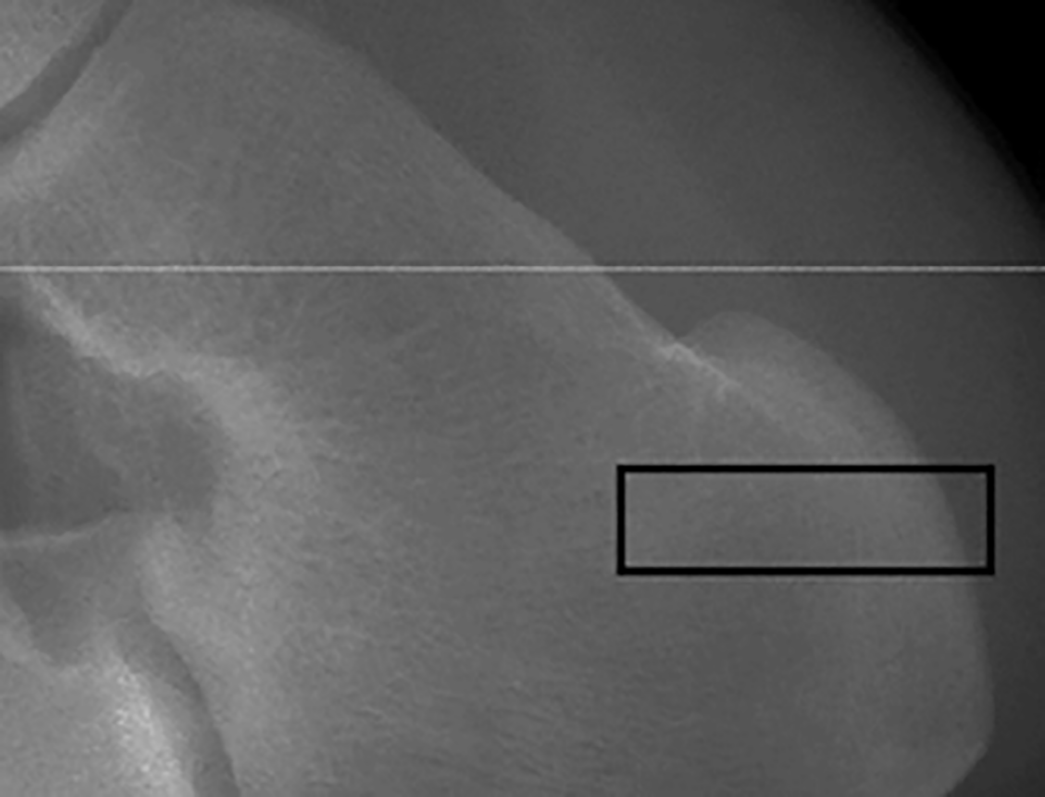
Calcaneus region of interest. Approximate calcaneus region of interest, sagittal view.

**Fig 2:**
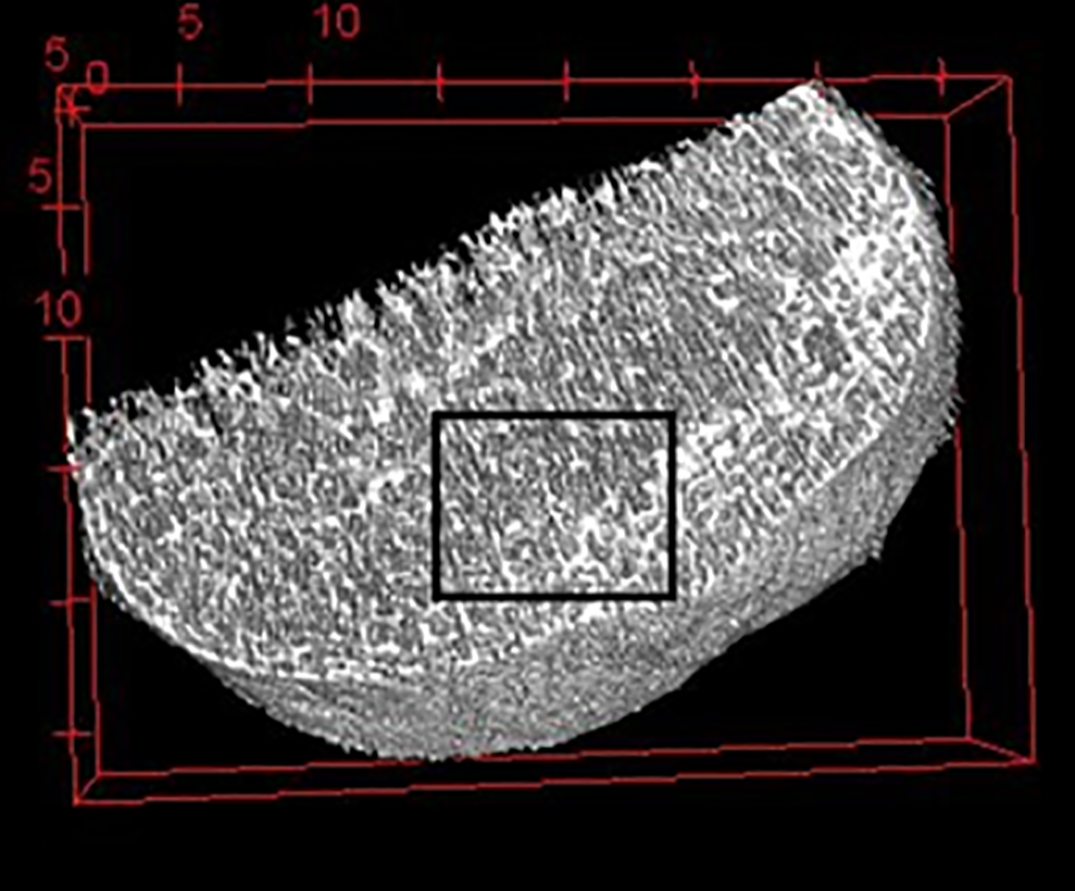
Calcaneus degree of anisotropy region of interest. Approximate calcaneus degree of anisotropy region of interest, superior view.

We focused our calcaneus analysis to a region of interest (ROI) located in the Achilles tuberosity, in the posterior region of the bone. This region is subject to high Achilles tendon forces and has been found to contain the greatest number of trabeculae in the calcaneus [33], thus we considered it a promising region for investigating the effects of endurance running. We defined this ROI as starting 15mm inferior to the talo-calcaneal joint and extending 9mm inferiorly, abutting the cortical bone of the calcaneal tuberosity and encompassing the entire medial-lateral breadth of the bone (Fig 1). This ROI was used in the analysis of all variables except degree of anisotropy (DA). DA analysis required defining an additional, smaller ROI within the initial ROI. This region was defined as an 8x8x9mm box abutting the cortical bone of the calcaneal tuberosity in the superior-most slices, and because the bone extends more proximally as we panned through the image stacks, the ROI becomes more proximal (farther from the cortical bone) in the inferior slices (Fig 2) Degree of anisotropy was analyzed in BoneJ, which as previously published uses the mean intercept length method [8]. This procedure was repeated for a total of three measures per participant to improve precision; reported DA values are three-measure averages.

To obtain CT images, each subject’s right foot was immobilized in a carbon fiber brace and scout scans were taken which were used to locate the ROI’s. Calcaneal image stacks were pared down for some participants to edit out regions of bone we did not intend to sample; this resulted in several calcaneal scans less than 9mm thick. Stacks of 110 images were reconstructed from each calcaneus scan to create 3D 9mm-thick volumes. Scanco software was used to measure Tb.N, Tb.Th, BV/TV, trabecular spacing (Tb.Sp, a function of Tb.N and Tb.Th) and DTrab. Before calculating trabecular properties, cortical and trabecular bone were automatically separated by Scanco software using a threshold-based algorithm with the threshold set to one third the apparent cortical bone density (see Laib et al., 1998 for a detailed explanation of this method). Degree of anisotropy was measured using the BoneJ plugin [45] for ImageJ v 1.48. Values close to 1 indicate anisotropic alignment while values closer to 0 indicate isotropic alignment of trabeculae. BoneJ is unable to separate cortical from trabecular bone. As such, we defined ROI’s within the calcaneus as previously discussed.

### Statistical analysis

All statistical analyses for trabecular measures were performed using SPSS version 22 statistical software (IBM). Trabecular measures obtained from CT scans were compared between RFS, FFS and non-runner groups using ANOVAs with Tukey *post hoc* tests, and between runners and nonrunners with t-tests. To explore the effects of covariates (age, body mass, weekly running distance, years running, and age at onset of running) we performed linear regressions for each covariate vs. each trabecular variable for runners and nonrunners separately. Where significant correlations were found between covariates and trabecular variables, ANCOVAs were performed to account for these effects. To inform our data interpretation given our small sample size, we computed Cohen’s *d* effect sizes (difference between the means of each group divided by the pooled standard deviation) for between-group differences using Lee Becker’s online effect size calculator [46]. Finally, we calculated *post hoc* observed power with SPSS ANOVA.

## Results

**Fig 3:**
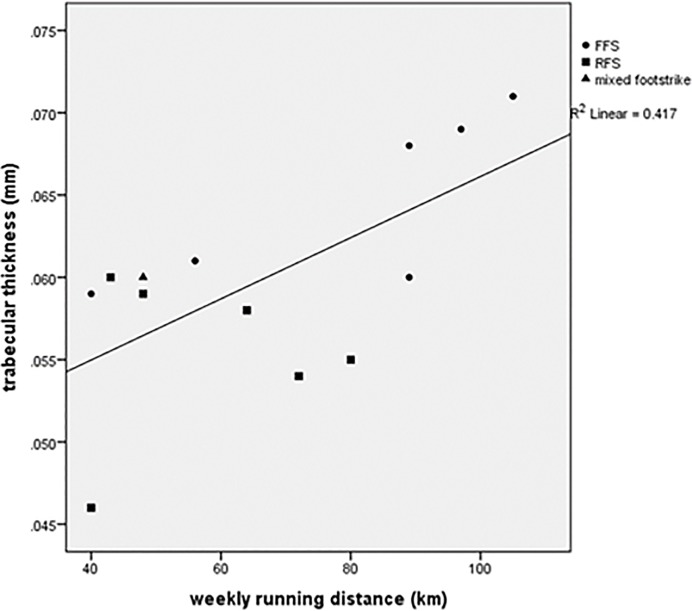
Trabecular thickness vs. weekly running distance.

**Fig 4:**
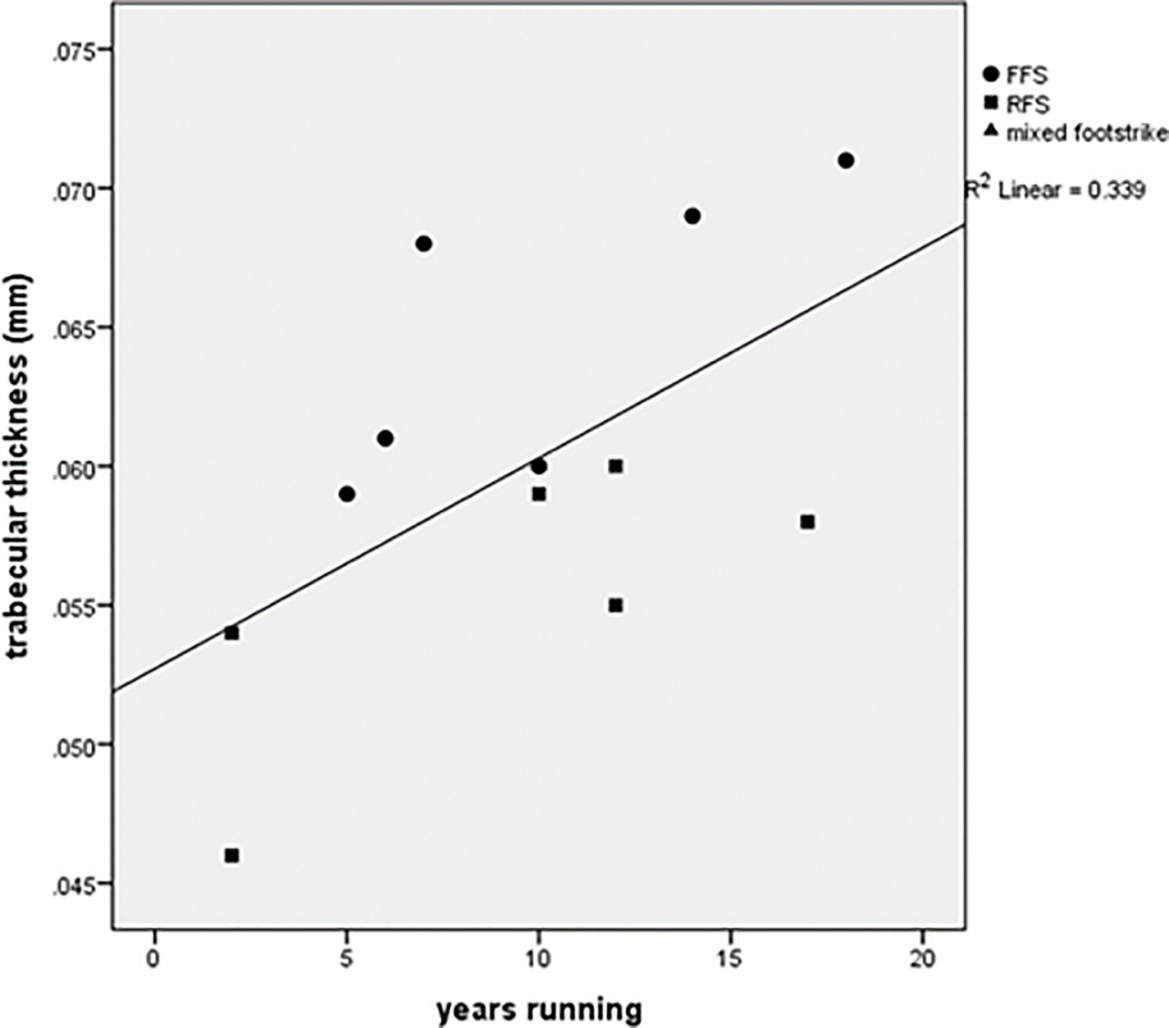
Trabecular thickness vs. years running.

**Fig 5:**
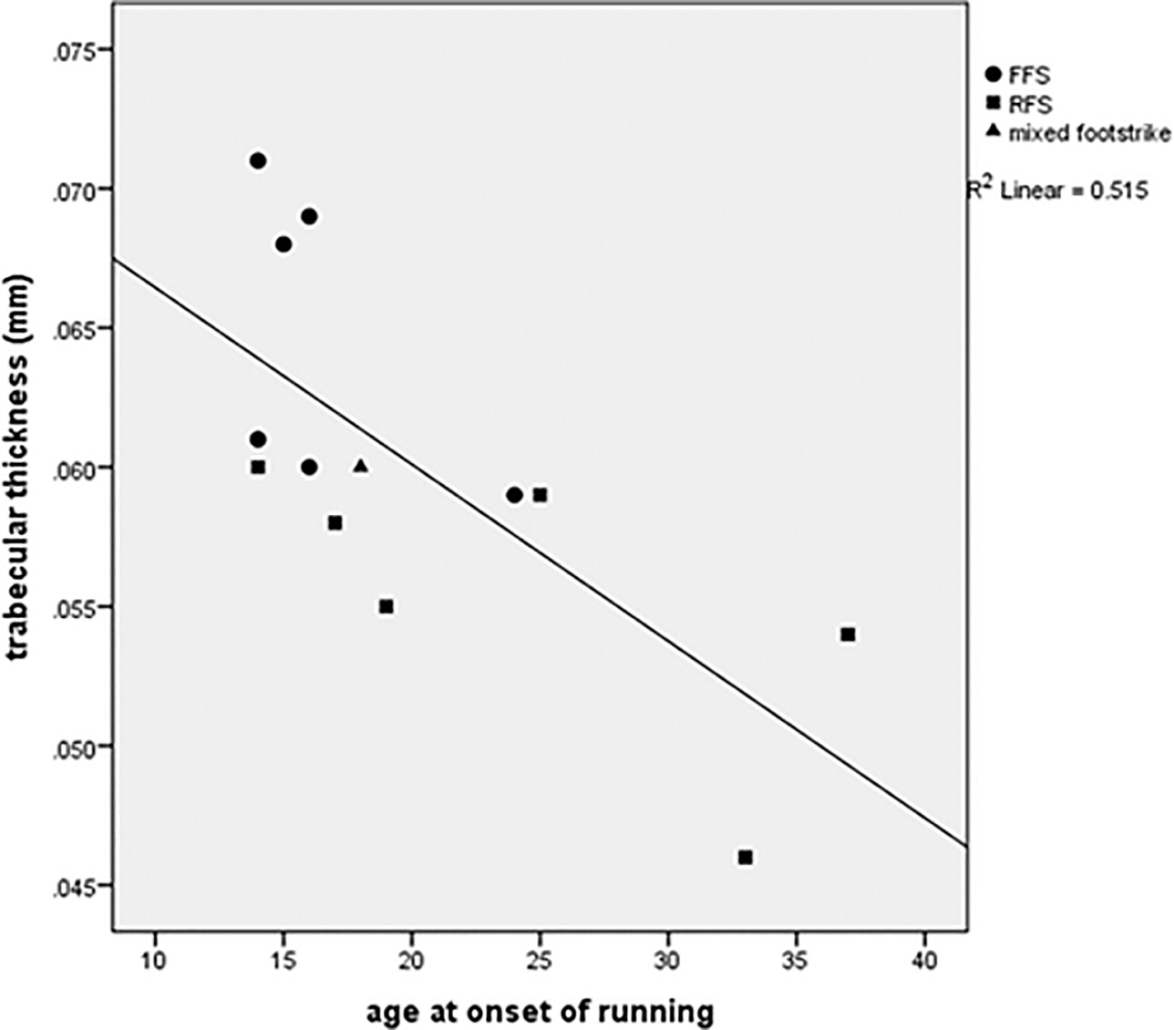
Trabecular thickness vs. age at onset of running.

There were no statistically significant intergroup differences in absolute trabecular variables though many had moderate (≥0.50) or large (≥0.80) effect sizes per Cohen [47] (Table 2). The largest effect sizes were found in comparisons of FFS vs. RFS, for three variables: Tb.Th (2.00), DTrab (1.17), and BV/TV (1.16). Several measures of trabecular robusticity- DTrab, Tb.Th and BV/TV- were highly correlated with body mass in nonrunners (r^2^ = 0.762, 0.824, and 0.757 respectively) (Table 3). These measures were poorly correlated with body mass in runners. In runners, Tb.Th was positively correlated with weekly running distance (r^2^ = 0.417, p<0.05) and years running (r^2^ = 0.339, p<0.05), and negatively correlated with age at onset of running (r^2^ = 0.515, p<0.01) (Figs 3–5). A regression incorporating these 3 variables generated an r^2^ value of 0.682 (p = 0.01). Adjusting Tb.Th values for years running revealed higher Tb.Th in FFS (p<0.01), but adjustment for all significant covariates rendered this difference insignificant (FFS Tb.Th: 0.063; RFS Tb.Th: 0.057; p = 0.11). Adjusting for body mass revealed greater Tb.Th in FFS than RFS (p<0.05) and nonrunners (p = 0.05). *Post hoc* observed power for ANCOVA’s ranged from 0.215 (Tb.Th adjusted for all significant covariates) to 0.878 (Tb.Th adjusted for years running) (Table 4).

**Table 2:** Trabecular properties of the posterior calcaneus.

Trab. measure	FFS mean (n = 6)	RFS mean (n = 6)	Runner mean (n = 13)	NR mean (n = 6)	Cohen’s *d* runners vs. NR	Cohen’s *d* FFS vs. RFS	Cohen’s *d* FFS vs. NR	Cohen’s *d* RFS vs. NR
DTrab (mg HA/cm^3^)	258.8 ±27.3	227.7 ±25.7	242.4 ±28.9	256.8 ±47.7	-0.37	1.17	0.05	-0.76
Tb.Th (mm)	0.065 ±0.005	0.055 ±0.005	0.060 ±0.007	0.061 ±0.011	-0.11	2.00	0.47	-.070
Tb.N (1/mm)	3.35 ±0.32	3.42 ±0.21	3.37 ±0.25	3.52 ±0.21	-0.65	-0.26	-0.63	-0.48
Tb.Sp (mm)	0.236 ±.029	0.238 ±0.018	0.238 ±0.022	0.225 ±0.021	0.60	-0.08	0.43	0.66
DA	0.551 ±.178	0.626 ±0.139	0.583 ±0.151	0.641 ±0.065	-0.50	-0.47	-0.67	-0.14
BV/TV	0.216 ±.023	0.190 ±0.022	0.202 ±0.024	0.214 ±0.040	-0.36	1.16	0.06	-0.74

Trabecular properties of the posterior calcaneus (mean±SD) with Cohen’s *d* effect sizes.

**Table 3:** Coefficients of determination (r^2^ values) for potential covariates vs. dependent variables.

Runners					
	DTrab	TbTh	TbN	DA	BV/TV
Age	0.230	0.199	0.007	0.006	0.231
Body mass	0.061	0.271	0.122	0.091	0.060
Weekly running distance	0.222	0.417*	0.027	0.088	0.218
Years Running	0.065	0.339*	0.177	0.134	0.066
Age at onset of running	0.269	0.515**	0.052	0.092	0.271
Nonrunners					
	DTrab	TbTh	TbN	DA	BV/TV
Age	0.110	0.072	0.065	0.063	0.111
Body mass	0.762*	0.824*	0.002	0.069	0.757*

Coefficients of determination (r^2^ values) for potential covariates vs. dependent variables.

*p<0.05.

**p<0.01.

**Table 4:** Results of ANCOVAs.

	FFS mean	RFS mean	Runner mean	NR mean	Observed power
*n*	6	6	13	6	
DTrab (mg HA/cm^3^) adjusted for body mass	274.4	233.5	251.2	237.8	0.522
Tb.Th (mm) adjusted for body mass	0.068*	0.057	0.062	0.056	0.779
Tb.Th (mm) adjusted for age at onset of running	0.063	0.057	—-	—-	0.323
Tb.Th (mm) adjusted for years running	0.064**	0.056	—-	—-	0.878
Tb.Th (mm) adjusted for weekly running distance	0.063	0.057	—-	—-	0.398
Tb.Th (mm) adjusted for body mass, age at onset of running, years running, and weekly running distance	0.064	0.056	—-	—-	0.215
BV/TV adjusted for body mass	0.215	0.191	0.201	0.198	0.243

*Greater than RFS (p<0.05) and nonrunners (p = 0.05).

**Greater than RFS (p<0.01).

## Discussion

Before body mass adjustment no trabecular variables were significantly different between test groups. Statistical power was likely insufficient to detect all intergroup differences. Nonetheless, we found evidence of bone adaptation in runners after accounting for effects of covariates and considering effect sizes. Body mass, which was significantly and substantially greater in nonrunners, predicted 82% of variation in Tb.Th and 76% of variation in DTrab and BV/TV of nonrunners but had little association with any trabecular variables in runners. An ANCOVA adjustment for body mass applied to all participants shows greater Tb.Th in FFS compared with nonrunners (p = 0.05) and RFS (p<0.05). Trabecular thickness was highly and significantly correlated with weekly running distance, years running, and age at onset of running, which together explain 68% of the variation in Tb.Th of runners. Those who run more and have been doing so for longer have thicker trabeculae, suggesting an unsurprising dose dependence in trabecular bone response. The inverse correlation between trabecular thickness and age at onset of running suggests increased plasticity of the bone modeling response earlier in life.

Given 1) a strong influence of loading history on Tb.Th; 2) earlier age at onset of running and greater running volume in FFS; and 3) our small sample size, it is difficult to discern between two competing explanations for greater Tb.Th (and perhaps DTtrab and BV/TV) in FFS. One explanation is that mass-adjusted Tb.Th in FFS and nonrunners may be an artifact of this group’s loading history: runners’ posterior calcaneal trabeculae become significantly thicker than that of nonrunners only with sufficient years and volume of running and footstrike has no observable effect. Alternatively, as Tb.Th, DTrab and BV/TV are still higher in FFS after adjusting for covariates (individually and collectively) and strong effect sizes are observed, footstrike may have an influence on trabecular adaptation in the posterior calcaneus. This could be resultant from greater Achilles tendon forces associated with forefoot striking as we hypothesized. It is likely that this study lacked sufficient power to detect significant differences in DTrab and BV/TV.

The correlation between body mass and Tb.Th observed here is fairly novel but perhaps not surprising. Across taxa spanning logarithmic differences in body mass, trabecular thickness scales with body mass but nowhere near isometrically [48, 49, 50, 51]; across small-bodied primates, however, vertebral Tb.Th scales isometrically with body mass [52]. To our knowledge no studies have identified an intraspecific body mass effect on trabecular thickness or mineral density in the human lower limb, though at least one study [23] used adjusted human trabecular variables based on nonhuman primate body mass/trabecular variable regressions. Additionally, a positive correlation between body weight and Tb.Th has been observed in the rat tibia [53]. Given that experimental results show increased Tb.Th as a result of loading, it stands to reason that body mass may have such an effect. Body mass was not correlated with any trabecular variables in runners, suggesting that forces producing during running trump daily locomotor and weight bearing forces that are primarily a function of body mass.

As we hypothesized, calcaneal anisotropy did not differ between any test groups. The region of the calcaneus that we sampled is subject to multiple forces and so contains trabeculae oriented in various planes. Endurance running presumably increases total loading in these planes and therefore we should not expect trabeculae to be highly aligned in one principal direction.

Contrary to our expectation, we did not observe significant differences in BV/TV, even after adjustment for body mass (though effect size was substantial for FFS vs. RFS). This may be an artifact of higher Tb.N in our nonrunner sample. Trabecular number is probably determined early in life and is not thought to respond vigorously as part of bone functional adaptation later in life- thus, higher Tb.N is our nonrunner sample probably results from sampling error. Because BV/TV is a function of Tb.Th and Tb.N, increased Tb.Th is the only likely way to increase BV/TV in an adult sample, and we observed no difference in Tb.Th without adjusting for body mass. Thus, BV/TV as a marker of bone adaptation is simply an indirect measure of Tb.Th.

In addition to having small sample sizes, our test groups were heterogeneous for variables that ideally would be kept relatively constant. Earlier mean age at onset of running and greater mean running volume in FFS confounds efforts to gauge the influence of footstrike on trabecular structure. Additionally, our nonrunner test group was comprised of individuals of varying activity levels and loading histories. While it is conceivable that this variation may have influenced nonrunners’ trabecular architecture, we intentionally recruited nonrunners who were at least slightly active; otherwise, we would essentially be comparing active vs. inactive participants rather than runners vs. nonrunners. To elucidate differences in trabecular adaptation resultant from walking vs. running, future research could include a test group comprised entirely of highly mobile nonrunners, such as backpackers.

## Conclusions

Our results add to the body of literature regarding locomotor-induced trabecular bone adaptation. The posterior region of the calcaneus appears to reflect loading resultant from endurance running, with trabecular thickness increasing in a dose dependent relationship. Perhaps because of an increased bone modeling response early in life, earlier onset of habitual endurance running was associated with greater trabecular thickness in the posterior calcaneus, suggesting that bone health may be improved through early adoption of load bearing exercise. Mean trabecular thickness, mineral density and bone volume fraction were greatest in FFS, but these differences were not statistically significant after adjustment for all covariates. Further research and larger sample sizes are needed to elucidate the role of footstrike in calcaneal trabecular robusticity.

## Supporting information

S1 Table Trabecular data. (XLSX)
