# Correction: Trabecular bone in the calcaneus of runners

**DOI:** 10.1371/journal.pone.0190553

**Published:** 2017-12-27

**Authors:** Andrew Best, Brigitte Holt, Karen L. Troy, Joseph Hamill

The third author’s name appears incorrectly. The correct name is: Karen L. Troy. The correct citation is: Best A, Holt B, Troy KL, Hamill J (2017) Trabecular bone in the calcaneus of runners. PLoS ONE 12(11): e0188200. https://doi.org/10.1371/journal.pone.0188200
